# Diagnostic efficacy of visual subtypes and low attenuation area based on HRCT in the diagnosis of COPD

**DOI:** 10.1186/s12890-022-01875-6

**Published:** 2022-03-06

**Authors:** Dan Zhu, Chen Qiao, Huiling Dai, Yunqian Hu, Qian Xi

**Affiliations:** 1 Department of Radiology, Shanghai East Hospital, Tongji University School of Medicine, No. 150 Jimo Road, Pudong New Area, Shanghai, 200120 China; 2 Department of Pulmonary and Critical Care Medicine, Shanghai East Hospital, Tongji University School of Medicine, Shanghai, China

**Keywords:** Chronic obstructive pulmonary disease (COPD), Visual subtypes, Low attenuation area (LAA), High-resolution computed tomography (HRCT), Diagnostic efficacy

## Abstract

**Background:**

Chronic obstructive pulmonary disease (COPD) is a heterogeneous disease. Current gold standard criteria, pulmonary function tests (PFTs) may result in underdiagnosis of potential COPD patients. Therefore, we hypothesize that the combination of high-resolution computed tomography (HRCT) and clinical basic characteristics will enable the identification of more COPD patients.

**Methods:**

A total of 284 patients with respiratory symptoms who were current or former smokers were included in the study, and were further divided into 5 groups of GOLD grade I–IV and non-COPD according to PFTs. All patients underwent inspiratory HRCT scanning and low attenuation area (LAA) was measured. Then they were divided into seven visual subtypes according to the Fleischner Society classification system. Non-parametric tests were used for exploring differences in basic characteristics and PFTs between different groups of enrolled patients and visual subtypes. Binary logistic regression was to find the influencing factors that affected the patients’ outcome (non-COPD vs GOLD I-IV). The area under the receiver operating characteristic curve (AUC-ROC) was to explore the diagnostic efficacy of LAA, visual subtypes, and combined basic characteristics related to COPD for COPD diagnosis. Finally, based on the cut-off values of ROC analysis, exploring HRCT features in patients who do not meet the diagnostic criteria but clinically suspected COPD.

**Results:**

With the worsening severity of COPD, the visual subtypes gradually progressed (*p* < 0.01). There was a significant difference in LAA between GOLD II–IV and non-COPD (*p* < 0.0001). The diagnostic efficacy of LAA, visual subtypes, and LAA combined with visual subtypes for COPD were 0.742, 0.682 and 0.730 respectively. The diagnostic efficacy increased to 0.923–0.943 when basic characteristics were added (all *p* < 0.001). Based on the cut-off value of ROC analysis, LAA greater than 5.6, worsening of visual subtypes, combined with positive basic characteristics can help identify some potential COPD patients.

**Conclusion:**

The heterogeneous phenotype of COPD requires a combination of multiple evaluation methods. The diagnostic efficacy of combining LAA, visual subtypes, and basic characteristics achieves good consistency with current diagnostic criteria.

## Background

Chronic obstructive pulmonary disease (COPD) is a common, preventable, and treatable heterogeneous disease with diverse clinical manifestations [1]. The incidence of COPD increases each year, and it is estimated that by 2030, nearly 4.5 million people will die from COPD and related diseases annually; by 2060, more than 5.4 million will die annually [2, 3]. This status quo has placed considerable pressure on society. Thus, current research is focused on the accurate identification of patients with COPD. Since the late 1990s, the diagnosis of COPD has been based on the Global Initiative for Chronic Obstructive Lung Disease (GOLD) criteria: diagnosis of COPD is confirmed when in the presence of risk factor exposure and respiratory symptoms, after inhaling bronchodilators, the forced expiratory volume in 1 s per forced vital capacity (FEV1/FVC) is less than 70% [4, 5]. Pulmonary function tests (PFTs) are the mainstay for establishing diagnoses, grading disease severity, evaluating treatment response, and predicting mortality in patients with COPD [6, 7]. However, in recent years, studies on the COPD gene have found that the current definition of the disease is too narrow; therefore, researchers have searched for more suitable methods to identify those who do not conform to PFTs but are at risk or have symptoms of COPD and try to develop new diagnostic criteria [8–10].

The pathogenesis of COPD is complex. Smoking, heredity, and biofuel exposure are involved in its pathogenesis [11]. Although radiography is not typically used in the diagnosis or management of COPD, it can reveal characteristics of bronchitis and emphysema in COPD patients, which are pathological bases of COPD [12]. COPD is frequently diagnosed and staged using PFTs based on the GOLD diagnostic criteria. However, PFTs cannot distinguish different pathophysiological mechanisms of COPD, which include emphysema, small airway destruction, and airway inflammation [13, 14]. In contrast, high-resolution computed tomography (HRCT) enables the quantification of abnormal changes in the lung parenchyma, including emphysema and bronchitis [15–17]. Moreover, COPD can be divided into different imaging phenotypes according to HRCT, and visual subtypes are often used for radiological diagnoses of emphysema [18]. The quantification of emphysema can be achieved by applying post-processing software. Low attenuation area (LAA) refers to the percentage of lung density that is below a certain threshold. Generally, − 950 Hounsfield Units (HU) is the threshold used to assess the severity of emphysema [19]. LAA and visual subtypes may help to identify patients with COPD symptoms with negative PFTs but who have positive imaging findings [12, 14]. In this article, we divided COPD into seven visual subtypes according to the Fleischner Society classification system and calculated LAA percentage by post-processing according to HRCT findings. We aimed to study the differences of basic clinical characteristics and PFTs among enrolled patients with different visual subtypes and COPD grades. Then, we further explored the diagnostic efficacy of LAA and visual subtypes based on the current diagnostic criteria of COPD. In addition, we used binary logistic regression to explore the influencing factors of COPD in these basic clinical characteristics, and further combined these influencing factors which had impact on COPD outcomes with LAA and visual subtypes to explore the combined diagnostic efficacy.

## Methods

### Subjects

We recruited patients who were current and former smokers attended Shanghai East Hospital from May 2020 to July 2021 because of cough, shortness or difficulty breathing. Patients provided writing informed consent before their examination, and all information obtained for the research was kept confidential. All enrolled participants underwent a physical examination, medical history was taken, PFTs were conducted, and chest HRCT scanning was performed. Inclusion criteria were patients aged ≥ 18 years with respiratory symptoms who smoked. Exclusion criteria were: (1) pregnant women; (2) other pulmonary diseases, such as asthma, bronchiectasis, pulmonary fibrosis, atelectasis, pulmonary infectious disease, active pulmonary tuberculosis, pleural effusion, and pneumothorax; (3) severe renal insufficiency, severe liver disease, human immunodeficiency virus, or other immune-related diseases; (4) confirmed or highly suspected lung cancer; (5) previous chest surgery; (6) severe cardiac insufficiency; (7) contraindication to salbutamol use; (8) in the acute phase of COPD. Finally, a total of 284 patients were included, which included 215 males and 69 females, ranging in age from 31 to 90 years. We collected basic information from all patients, which included age, sex, height, weight, history of exacerbations and symptoms, and conducted a questionnaire survey to determine the degree of dyspnea of all patients (modified British medical research council, mMRC).

### Imaging techniques

All participants underwent inspiratory HRCT with a Canon 320-detector row computed tomography (CT) scanner in the radiology department. Before scanning, patients performed deep inhalation training. A whole-lung scan was acquired while patients were in a supine position with both arms raised. Scanning parameters were: tube voltage 120 kV, tube current 50 mA, collimation 40 mm, pitch 1.0875, speed 0.275 r/s, layer thickness 1 mm, and matrix 512 × 512. The standard algorithm was used for reconstruction. Data with large artifacts and poor coordination were removed. Finally, data were processed using the Canon 320-detector row CT scanner post-processing software workstation.

### PFTs

All patients underwent PFTs after admission using the Jaeger Pulmonary Function Instrument from Germany (MasterScreen Diffusion). During the examination, patients were seated, inhalation and exhalation were carried out under the guidance of the technician. The main measurement indices were FEV1, FEV1/FVC, FEV1 percentage in predicted value (FEV1pred%), FVC, residual air volume (RV), total lung volume (TLC), and percentage of residual air volume in total lung volume (RV/TLC%). Participants were classified as COPD or non-COPD according to the 2019 GOLD guidelines [4]. COPD patients were further staged I to IV according to FEV1pred%: GOLD I (FEV1pred% ≥ 80%), GOLD II (50% ≤ FEV1pred% < 80%), GOLD III (30% ≤ FEV1pred% < 50%), GOLD IV (FEV1pred% < 30%) [20].

### Visual subtypes

**Fig. 1:**
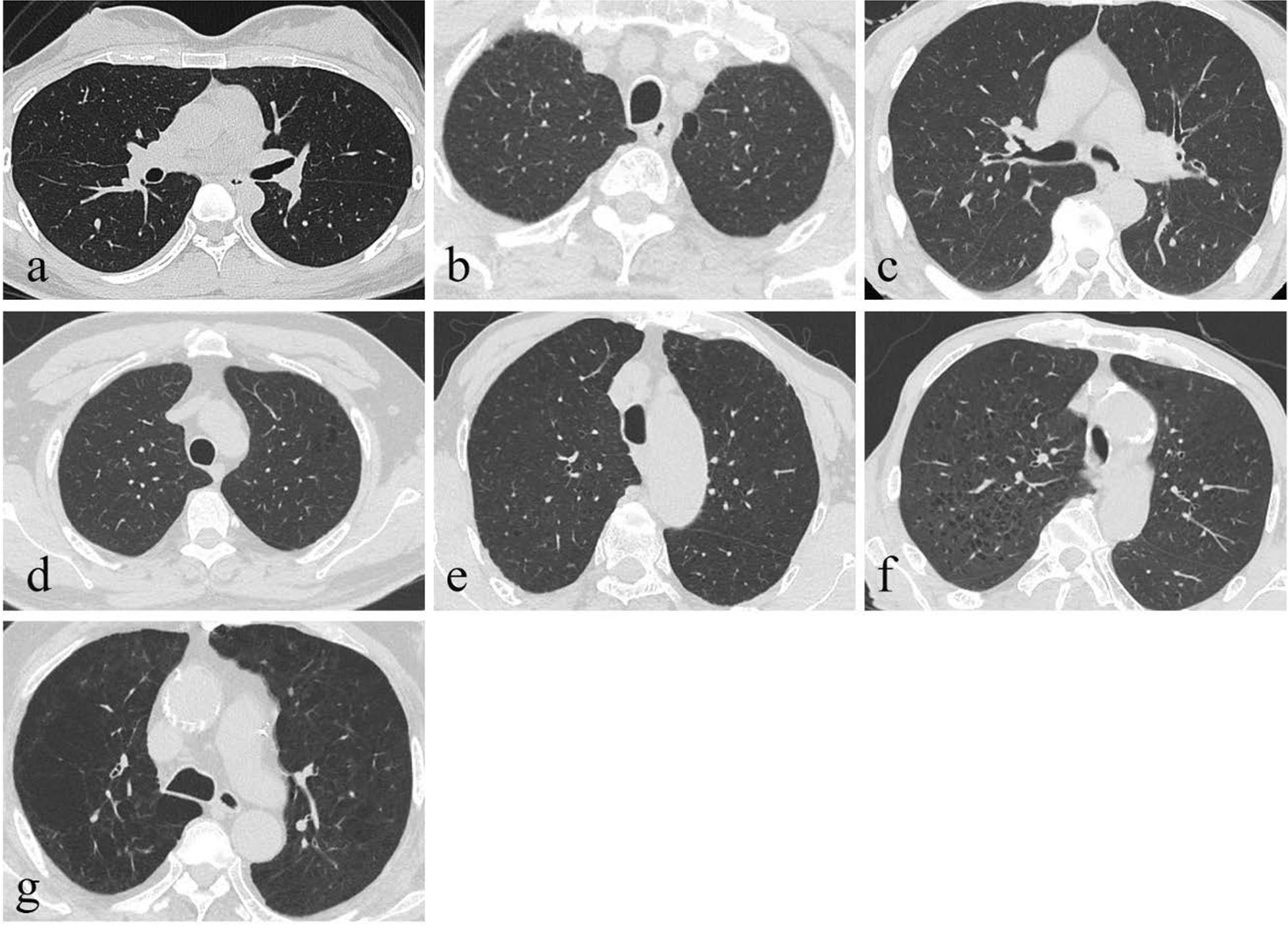
HRCT manifestations of seven different visual subtypes. **a** Normal. **b** Paraseptal emphysema. **c** Bronchitis; HRCT shows thickening of the bronchial wall without obvious emphysema area. **d** Centrilobular emphysema (mild); LAA accounts for less than 0.5%. **e** Centrilobular emphysema(moderate); LAA accounts for 0.5–5%. **f** Centrilobular emphysema (severe); LAA accounts for more than 5%. **g** Advanced destructive emphysema; Panlobular emphysema with bronchus hyperexpansion and distortion

We divided enrolled patients into the following seven visual subtypes: normal, paraseptal emphysema (PSE), bronchitis, centrilobular emphysema (mild, moderate, or severe), and advanced destructive emphysema based on Fleischner Society and inspiratory HRCT [20, 21]. Mild (0.5%), moderate (0.5%–5%), or severe (> 5%) centrilobular emphysema was classified according to the percentage of emphysema area in the whole lung. Advanced destructive emphysema referred to panlobular emphysema with bronchus hyperexpansion and distortion. Some researchers had studied the relationship between these seven subtypes with PFTs and indicated that the degree of lung damage of these seven subtypes were progressively aggravated, therefor they could be regarded as rank variables in terms of data types in the follow-up research in a certain extent. We first set visual subtypes as dummy variable in the binary logistic regression analysis, calculated the predicted probability, and finally performed the ROC analysis based on the predicted probability. Images of these seven visual subtypes were shown in Fig. 1. All visual subtypes were independently evaluated by two radiologists with 3 and 8 years of experience, respectively, without knowledge of patients’ clinical history. If there were disagreements, the two radiologists discussed until consensus was reached.

### Quantitative index measurement

**Fig. 2:**
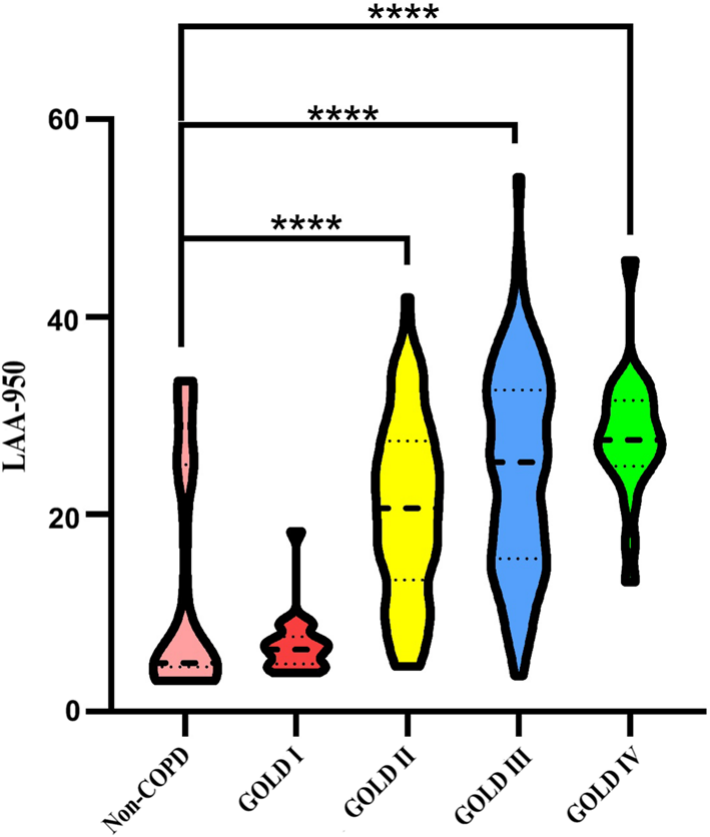
Violin plot of LAA-950 changes in 284 enrolled patients with different groups. The middle line represents the median, while the upper and lower lines represent the 25th and 75th quartile respectively. ****p < 0.0001

LAA-950 referred to the percentage of lung density less than -950 HU, which was automatically calculated by the Canon 320-detector row CT post-processing workstation. Figure 2 showed the distribution of LAA in different groups of 284 patients.

### Statistical analysis

We used IBM SPSS 25.0, MedCalc V19.4.1 and GraphPad Prism (version 8.0) to analyse the data. Firstly, we used Kolgomorov–Smirnov test to analyse the normality of the distribution, meanwhile, the homogeneity of variance was also verified. Continuous variables with skewed distribution were shown as median and interquartile ranges (IQR), while categorical variables were expressed as percentages. Groups were compared using the Kruskal–Wallis test for both of the GOLD groups and seven different visual subtypes were independent of each other. Then we used posterior comparisons to compare the differences between any two groups. The Kappa inter-rater reliability coefficient was used to evaluate the consistency of the visual analysis results between the two doctors. Subsequently, we divided the enrolled patients into two groups based on the COPD diagnostic gold criteria, the COPD group (GOLD I–IV) and the non-COPD group, and further explored correlations between basic characteristics and COPD by binary logistic regression. We used a step-forward technique (Wald test) and removed variables from the final model with *p* > 0.1. Odds Ratios (ORs), 95% confidence intervals (95% CI), and the probability of prediction were calculated simultaneously. Finally, we based on the probability of prediction calculating the area under the receiver operating characteristic curve (AUC-ROC) to determine the diagnostic efficacy of LAA, visual subtypes, and the combination of the two for COPD. And then, combining the basic characteristics of enrolled patients which was associated with COPD with LAA and visual subtypes respectively to obtain combined diagnostic efficacy. Finally, based on the cut-off values of ROC analysis, exploring HRCT features in patients who do not meet the diagnostic criteria but clinically suspected COPD. In this article, *p* < 0.05 was considered statistically significant.

## Results

According to COPD diagnosis and severity grading criteria, 83 patients were non-COPD, 18 patients were GOLD I, 82 patients were GOLD II, 80 patients were GOLD III, and 21 patients were GOLD IV. Detailed basic characteristics of patients with different groups were shown in Table 1. Among the 284 patients, GOLD grades I and IV were the least common. This is in line with the current status quo, where early and late COPD patients are not easily detected by current COPD diagnostic criteria. In addition, there were significant differences in age, gender, body mass index (BMI), mMRC score, acute exacerbation frequency and PFTs between enrolled patients of different groups (all *p* < 0.01), which is consistent with previous findings [22–24]. The violin plot showed that with the increase in severity of COPD, the value of LAA-950 increased gradually (shown in Fig. 2). There was no difference between GOLD grade I with non-COPD patients (*p* = 0.56 > 0.05), whereas a significant difference was observed between GOLD grades II to IV and non-COPD patients (all *p* < 0.0001).

**Table 1:** Demographic and clinical characteristics of different groups of enrolled patients

	Non-COPD	GOLD I	GOLD II	GOLD III	GOLD IV	*p* value
Number, N (%)	83 (29.26%)	18 (6.33%)	82 (28.87%)	80 (28.17%)	21 (7.39%)	–
Age, year	67 (59,68)	59.50 (55.75,65)	67.50 (60,76.25)	66.50 (62.25,71)	69 (67,69)	< 0.01
Sex, man, N (%)	43 (51.81%)	14 (77.78%)	68 (82.93%)	71 (88.75%)	19 (90.47%)	< 0.01
BMI, kg/m^2^	28.65 (25.80,29.75)	23.72 (23.29,24.52)	24.04 (21.60,25.68)	23.17 (21.03,26.01)	18.69 (17.98,19.60)	< 0.01
Current smoker, N (%)	49 (59.04%)	10 (55.56%)	67 (81.71%)	59 (73.75%)	18 (85.71%)	–
Acute exacerbations (frequency/year)	0 (0,1)	0 (0,1.5)	1 (1,3)	3 (1,4)	3.5 (2.75,4)	< 0.01
FEV1	1.56 (1.21,1.68)	2.54 (2.50,2.54)	1.62 (1.39,1.91)	1.11 (0.96,1.28)	0.78 (0.66,0.92)	< 0.01
FVC	2.45 (2.06,2.85)	3.70 (3.70,3.75)	2.58 (2.30,3.08)	2.09 (1.88,2.37)	1.57 (1.35,1.93)	< 0.01
FEV1%	53.20 (41.20,58.30)	81.00 (81.00,83.00)	57.10 (52.90,66.40)	39.80 (35.93,44.18)	26.70 (22.80,28.65)	< 0.01
FEV1/FVC%	61.11 (57.35,71.36)	67.79 (66.11,68.65)	62.92 (58.44,67.22)	51.65 (48.01,58.34)	50.00 (45.16,56.99)	< 0.01
RV/TLC%	52.79 (49.81,63.84)	39.81 (38.47,56.70)	55.85 (46.97,61.99)	60.90 (54.11,65.34)	66.41 (62.27,71.26)	< 0.01
LAA-950	4.90 (4.50,27.50)	6.25 (4.80,7.58)	20.60 (13.38,27.43)	25.30 (15.53,32.58)	27.50 (24.90,31.50)	< 0.01
Visual subtypes	3 (2,5)	2 (1.75,4)	4 (4,5)	5 (5,6)	6 (6,6)	< 0.01

Data were presented as median (interquartile range), absolute values and percentage

*FEV1* forced expiratory volume at 1S, *FVC* forced vital capacity; *FEV1%* FEV1 percentage in predicted value, *RV/TLC%* residual air volume in total lung volume percentage

The basic characteristics of enrolled 284 patients for the seven different visual subtypes based on Fleischner Society classification system were shown in Table 2. The inter-rater reliability coefficient kappa value of the visual subtypes determined by the two doctors was 0.875 (*p* < 0.001). Among the seven subtypes, advanced destructive emphysema was the least common, followed by the normal subtype, and patients of advanced destructive emphysema subtype tended to be older. The most common visual subtype in patients with non-COPD was bronchitis. Of the patients with GOLD grade I, the PSE subtype was the most common. Moreover, as the severity of COPD worsened, the visual subtypes gradually progressed (*p* < 0.01). BMI decreased significantly as the degree of emphysema worsened (*p* < 0.01). For mMRC scores, the first six subtypes showed no difference, whereas those with advanced destructive emphysema had the highest scores. In patients with normal CT findings, most patients’ mMRC score was three points. In addition, patients who smoked showed symptoms of cough, expectoration, and difficulty breathing. Regardless of subtype, difficulty breathing was the most common symptom, and patients with severe centrilobular emphysema and advanced destructive emphysema accounted for 96.05% and 87.50% of patients, respectively. As for acute exacerbation frequency, differences also existed between different visual subtypes (*p* < 0.01). The median (IQR) of PFTs of each subtype was listed in Table 2, the results showed there were no differences in PFTs between normal, PSE, and bronchitis subtypes. Subsequently, greater severity of emphysema was related to poorer PFTs (*p* < 0.01).

**Table 2:** Demographic grouped by visual subtypes by two different radiologists

Subtypes	Normal	PSE	Bronchitis	Centrilobular emphysema (mild)	Centrilobular emphysema (moderate)	Centrilobular emphysema (severe)	Advanced destructive emphysema	*p* Value
Number, N (%)	28 (9.86%)	31 (10.92%)	29 (10.21%)	49 (17.25%)	63 (22.18%)	76 (26.76%)	8 (2.82%)	–
Age, year	59 (58.25,64.75)	64 (56,68)	67 (58,81)	66 (59,75.5)	67 (64,73)	69 (63.25,72)	68.50 (64,78.75)	< 0.01
Sex, man, N (%)	17 (60.71%)	18 (58.06%)	14 (48.28%)	40 (81.63%)	57 (90.48%)	62 (81.58%)	7 (87.5%)	< 0.01
BMI, kg/m^2^	25.43 (23.49,29.38)	29.75 (24.80,30.00)	27.43 (24.68,29.75)	24.49 (21.97, 26.32)	24.80 (21.97, 25.86)	22.59 (18.98,25.33)	19.84 (17.28,21.88)	< 0.01
mMRC scores	3 (0,3)	2 (2,3)	2 (1,3)	2 (1,2)	2 (1,4)	3 (3,4)	4 (3,4)	< 0.01
Symptoms								< 0.01
Cough	5 (17.86%)	7 (22.58%)	6 (20.69%)	14 (28.57%)	10 (15.87%)	2 (2.63%)	0 (0%)	–
Expectoration	4 (14.29%)	11 (35.48%)	2 (6.90%)	8 (16.33%)	9 (14.29%)	1 (1.32%)	1 (12.50%)	–
Difficult breathing	19 (67.86%)	13 (41.94%)	21 (72.41%)	27 (55.10%)	44 (69.84%)	73 (96.05%)	7 (87.50%)	–
Current smoker, N (%)	16 (57.14%)	20 (64,52%)	25 (86.21%)	30 (61.22%)	51 (80.95%)	64 (84.21%)	6 (75%)	–
Acute exacerbations (frequency/year)	0 (0,1)	0 (0,1)	0 (0,2)	1 (0,2)	1 (0,2)	2 (1,3.5)	3.5 (2,4)	< 0.01
FEV1	1.46 (1.21, 1.84)	1.56 (1.21,2.50)	1.56 (1.21,1.89)	1.51 (1.06,1.95)	1.42 (1.17,1.91)	1.26 (0.90,1.51)	1.04 (0.74,1.21)	< 0.01
FVC	2.83 (2.24, 3.61)	2.45 (2.06, 3.70)	2.45 (1.98, 2.99)	2.40 (1.80, 3.15)	2.40 (2.04, 2.65)	2.27 (1.91, 2.65)	2.08 (1.54, 2.33)	< 0.01
FEV1%	47.25 (41.20, 63.95)	60 (41.20, 78.90)	58 (47.30, 68.00)	53.20 (45.35, 66.25)	51.40 (40.50, 58.30)	42 (31.98, 53.20)	32.85 (26.63, 44.70)	< 0.01
FEV1/FVC%	61.11 (60.96, 68.79)	63.67 (58.74,67.15)	62.13 (52.85,71.29)	63.21 (58.64,67.64)	57.35 (51.54, 67.00)	51.52 (46.78,59.74)	51.26 (46.60, 52.90)	< 0.01
RV/TLC%	49.81 (44.76, 50.62)	49.81 (49.81, 54.64)	52.79 (50.27, 62.47)	52.64 (46.29, 63.15)	59.23 (52.48, 67.28)	62.58 (57.01, 69.60)	63.33 (59.61, 69.46)	< 0.01

Data were presented as median (interquartile range), absolute values and percentage

*FEV1* forced expiratory volume at 1S, *FVC* forced vital capacity, *FEV1%* FEV1 percentage in predicted value, *RV/TLC%* residual air volume in total lung volume percentage

**Fig. 3:**
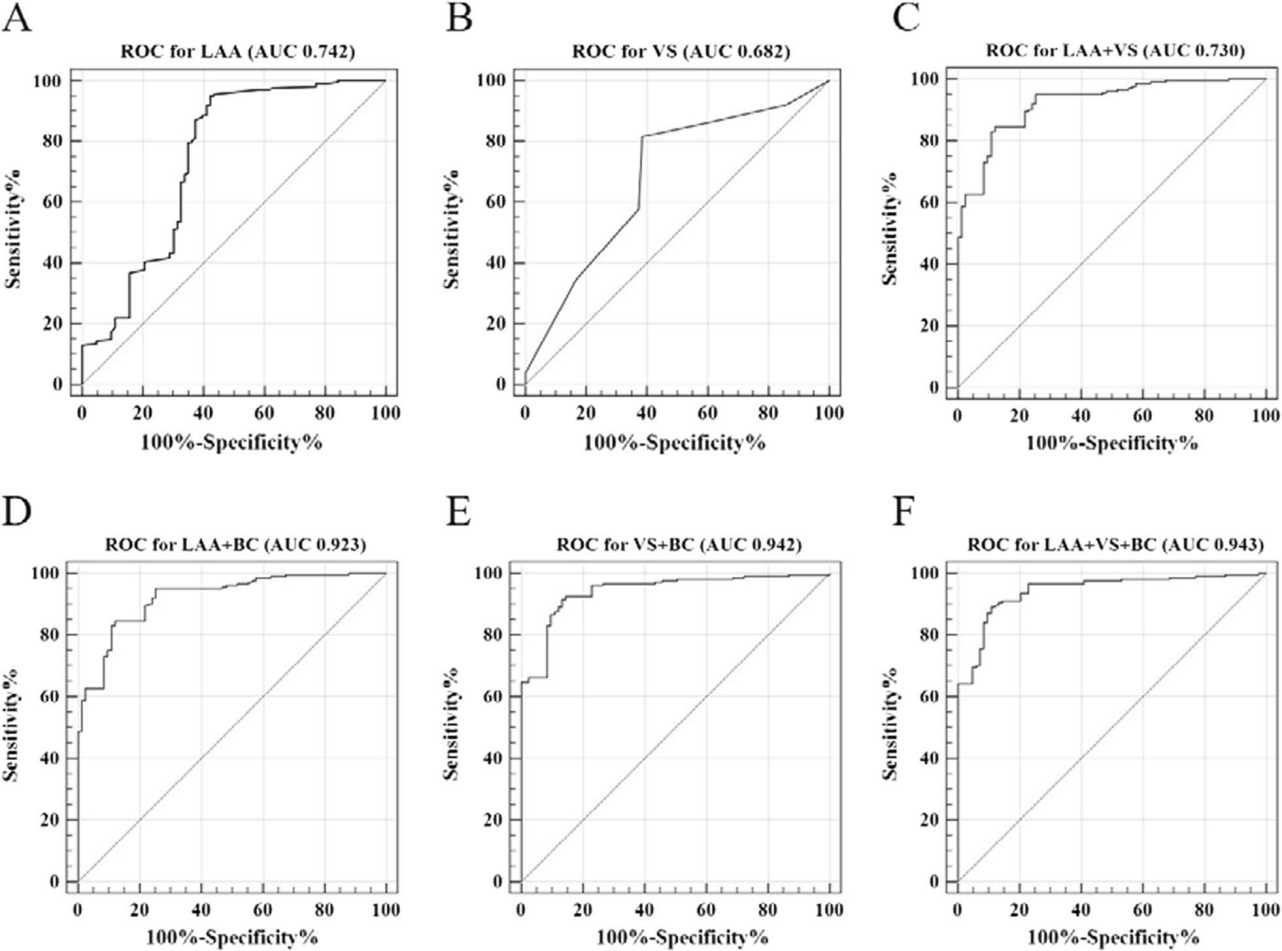
ROC curve for different methods on the diagnostic efficacy of COPD. **A**–**C** represented diagnostic efficiency of LAA, VS, LAA + VS respectively. **D**, **E** represented above indexes combined with basic characteristic in diagnosing COPD respectively. The plot showed that combining basic characteristics improved the diagnosis efficacy. *Notes*: *LAA* low attenuation area; *VS* visual subtypes, *BC* basic characteristics including symptoms, sex, BMI, mMRC scores and history of exacerbations

Finally, from binary logistic regression analysis results, basic characteristics (symptoms, sex, BMI, mMRC scores and history of exacerbations) were interrelated with COPD (all *p* < 0.001; Table 3). We found that age was not significantly related to COPD (*p* = 0.87 > 0.05). Therefore, age was not included in the basic characteristics in the later ROC analysis. Then, we studied the diagnostic efficacy of LAA-950 and the visual subtypes for COPD separately according to the current gold standard for COPD diagnosis. Visual subtypes and LAA-950 for diagnosis of COPD achieved an AUC-ROC of 0.682 (Z Value: 5.132, 95% CI: 0.625–0.736) and 0.742 (Z Value: 6.465, 95% CI: 0.687–0.792), respectively. Moreover, the combination of LAA-950 and visual subtypes achieved an AUC-ROC of 0.730 (Z Value: 6.133, 95% CI: 0.674–0.781). Then we incorporated basic characteristics into the ROC curve analysis, which resulted in a significantly higher AUC-ROC than that of the previous individual approaches (0.923–0.943; *p* < 0.001), of which LAA, visual subtypes combined with basic characteristics reached the highest AUC-ROC. The diagnostic efficacy, sensitivity, specificity and cut-off value of different methods were shown in Table 4. And Fig. 3 presented area under the curve (AUC) of different methods for diagnosis of COPD. From which we could see the specificity of the COPD diagnosis by LAA and visual subtypes alone was not high (57.8%–61.4%; *p* < 0.001). After adding basic characteristics, the specificity increased in varying degrees (86.75%–89.16%; *p* < 0.001). Finally, the pairwise comparisons among the six methods showed that the AUC-ROC of any two groups for diagnosing COPD was significantly different (all *p* < 0.001). In addition, the cut-off value for diagnosing COPD by LAA was 5.6, at the same time, centrilobular emphysema and advanced destructive emphysema were more indicative of COPD. Based on the cut-off values of ROC analysis, for some patients who are highly suspected of COPD but do not meet the diagnostic criteria of PFTs, when the LAA is greater than 5.6, and the visual subtype is greater than bronchitis subtype (that is visual subtype with centrilobular emphysema and advanced destructive emphysema), combined with positive basic characteristics of the patient, such as smoking history, sex of male, mMRC scores, low BMI, history of acute exacerbation, etc., the patient is more likely to suffer from COPD.

**Table 3:** Binary logistic regression of COPD influencing factors

Predictor	OR	95% CI	*p* Value
Symptoms			0.006
Cough (reference category)			
Shortness of breath	1.85	1.36–14.76	0.002
Difficulty breathing	2.23	1.02–5.75	< 0.001
Sex	5.52	3.08–9.89	< 0.001
mMRC scores			< 0.001
Level 0 (reference category)			
Level 1	4.42	0.95–20.62	0.059
Level 2	4.42	1.39–14.05	0.012
Level 3	1.88	1.40–1.93	0.007
Level 4	1.23	1.11–2.47	< 0.001
BMI, kg/m^2^	0.68	0.62–0.76	< 0.001
Age	1.00	0.98–1.03	0.875
History of exacerbations	3.94	2.30–6.75	< 0.001

*OR* odds ratio, *CI* confidence interval

**Table 4:** Diagnostic efficacy of different methods with COPD

Method	AUC-ROC	Sensibility%	Specificity%	Z value	95% CI	Cut-off Value	*p* Value
LAA	0.742	**95.02**	57.83	6.465	0.687–0.792	> 5.6	< 0.001
VS	0.682	81.59	61.45	5.132	0.625–0.736	> 3	< 0.001
LAA + VS	0.730	92.04	57.87	6.133	0.674–0.781	–	< 0.001
LAA + BC	0.923	84.58	87.95	26.350	0.886–0.951	–	< 0.001
VS + BC	0.942	91.54	86.75	31.779	0.908–0.966	–	< 0.001
LAA + VS + BC	**0.943**	89.05	**89.16**	32.601	0.910–0.967	–	< 0.001

The bold font is used to highlight the highest value of each column among each different method. Each row represents a different method

*LAA* low attenuation area, *VS* visual subtypes, *BC* basic characteristics including symptoms, sex, BMI and mMRC scores, history of exacerbations; *AUC* area under curve, *ROC* receiver operating characteristic, *CI* confidence interval

## Discussion

In our study, we divided HRCT manifestations of our patients into seven different visual subtypes based on the Fleischner Society classification system [20]. These seven subtypes exhibited significant differences in patient symptoms, age, sex, mMRC scores, acute exacerbation frequency and PFTs. Previous studies have also shown that visual subtypes of COPD were positively correlated with the risk of death [24]. These seven visual subtypes in the degree of lung damage were progressively aggravated to a certain extent, so, we had reasons to consider these seven visual subtypes as rank variables for further ROC analysis. Meanwhile, these results suggested that visual subtypes may can become a means of assisting the diagnosis of COPD, which provided a foundation for our follow-up research.

LAA is another method to assess the severity of COPD. LAA increases with the increasing COPD severity grade. The trapping of air in COPD patients results in reduced lung density. We found that among non-COPD patients, LAA-950 was greater than the previous standard of five in some patients, which indicated that these patients had imaging abnormalities that were insufficient for detection using PFTs. There was no significant difference between GOLD grade I and non-COPD patients (*p* = 0.56 > 0.05). The mismatch between LAA and PFTs indicated that relying on one method alone may be insufficient for the diagnosis of early COPD. In addition, centrilobular emphysema and advanced destructive emphysema was more indicative of COPD, which indicated that the current gold criteria more commonly identified patients with more serious illnesses. For patients with positive symptoms who exhibited imaging abnormalities, the sole reliance on PFTs may have deficiencies. However, there were also several patients with positive PFTs who had negative imaging findings. Therefore, additional methods may be needed to comprehensively evaluate these patients. Our findings suggested the potential of combining diagnostic methods to compensate for current deficiencies in diagnosis so that to discover more potential patients.

We demonstrated that HRCT may be useful for the diagnosis of COPD. Our study showed that visual subtypes and LAA offer diagnostic value for COPD based on current gold standard criteria, with AUC-ROCs of 0.742 and 0.682, respectively. The use of visual subtypes or LAA alone to diagnose COPD did not provide high diagnostic efficacy. Nevertheless, the matching with the gold standard indicated that these methods can accurately identify some COPD patients. The mismatching part may include patients who had poor pulmonary function but with normal FEV1/FVC. Such mismatch between imaging findings and PFTs may provide further insight into further development of diagnostic criteria for COPD.

COPD is a heterogeneous disease with a high rate of incidence [25]. Current diagnostic criteria of PFTs play a vital role in the diagnosis, severity assessment, and longitudinal monitoring of COPD [26]. However, PFTs only provide global information on lung damage, and the visualization of regional information is limited [10, 21]. Nevertheless, HRCT resolves these limitations to some extent and can be used to assess the type, extent, distribution, and progression of COPD [15, 27]. Previous studies have shown that quantitative CT analysis is correlated with PFT results and pathology-based quantification of emphysema [21, 27, 28]. Moreover, there are numerous reports using quantitative CT imaging in COPD patients, demonstrating its wide use as a simple imaging method to obtain information on the whole lung [29, 30]. Similarly, based on our research results, we found that LAA and visual subtypes were crucial in identifying COPD patients and assessing the severity of COPD patients. Binary logistic regression results showed that basic characteristics of symptoms, sex, BMI, mMRC scores and history of exacerbations were related to COPD. Men were 5.52 times more likely to develop COPD than women. Patients with a positive history of exacerbations were 3.94 times as likely as those with a negative history. In addition, the severity of the patient's symptoms and the increase of mMRC scores were both risk factors for the development of COPD. However, our study concluded that age was not a risk factor for COPD, so we excluded the age factor in the follow-up ROC analysis, which may be related to our relatively insufficient sample size. When these characteristics were combined with LAA and visual subtypes to evaluate COPD patients, the diagnostic efficacy reached 0.943. In addition, the combination of basic characteristics with LAA or visual subtypes, the diagnostic efficacy reached 0.923 and 0.942, respectively. These findings highlight the heterogeneity of COPD as a disease that is affected by multiple factors, and our prediction model achieved a higher consistency of COPD diagnosis than did the gold standard criteria. In future research, although it is not clear whether the basic characteristics of COPD, LAA, and visual subtypes can be used as diagnostic criteria for COPD, our study demonstrated that the evaluation of the heterogeneous phenotype of COPD required a combination of multiple methods. Furthermore, our model may be helpful in quantifying disease severity or identifying unique clinical subgroups prospectively. Therefore, in the future clinical work, for those patients with FEV1/FVC > 0.7, and cannot be diagnosed with COPD according to the current diagnostic gold criteria, we may need to pay attention to HRCT finding as well. There may be abnormalities on HRCT but not in the FEV1/FVC ratio. Currently, there was no basis for diagnosing them as COPD, but based on our study, it provided a possibility for combined diagnosis. Consequently, for those patients who do not meet the diagnostic criteria of PFTs, the abnormalities on HRCT should also be taken into account, and more basic characteristic should be combined to confirm the diagnosis, so as to get early treatment for these potential COPD patients.

Our study had several limitations. Firstly, the number of patients in each subtype was relatively small, especially those with the advanced destructive emphysema subtype. Secondly, some patients may conform to two of these subtypes. For example, patients with centrilobular emphysema subtype also had bronchitis, which we classified as the former subtype. Thirdly, our subjects were current and former smokers. Although smoking was a significant factor in the development of COPD, there were numerous other causes of COPD. Therefore, our findings may not apply to all patients.

## Conclusions

We found that the diagnostic efficacy of combining LAA, visual subtypes, and basic characteristics for COPD achieved good consistency with current diagnostic criteria. Our findings confirmed that COPD is a highly heterogeneous disease, and LAA and visual subtypes may be useful for describing this heterogeneity. Whether LAA and visual subtypes can be combined with PFTs for the diagnosis of COPD in the future remains unclear. Nevertheless, our study offers new avenues for combining measures to diagnose and detect more potential COPD patients.

## Data Availability

The datasets used and analysed during the current study are available from the corresponding author on reasonable request.

## Abbreviations

COPD: Chronic obstructive pulmonary disease
PFTs: Pulmonary function tests
HRCT: High-resolution computed tomography
BMI: Body mass index
FEV1: Forced expiratory volume at 1S
FVC: Forced vital capacity
FEV1%: FEV1 percentage in predicted value
RV/TLC%: Residual air volume in total lung volume percentage
LAA: Low attenuation area
GOLD: Global initiative for chronic obstructive lung disease
AUC: Area under curve
ROC: Receiver operating characteristic
